# Effectiveness of BNT162b2 and ChAdOx1 Vaccines against Symptomatic COVID-19 among Healthcare Workers in Kuwait: A Retrospective Cohort Study

**DOI:** 10.3390/healthcare9121692

**Published:** 2021-12-07

**Authors:** Walid Q. Alali, Lamiaa A. Ali, Mohammad AlSeaidan, Mohammad Al-Rashidi

**Affiliations:** 1 Department of Epidemiology & Biostatistics, Faculty of Public Health, Kuwait University, Kuwait City 13060, Kuwait; 2 Department of Public Health, Faculty of Medicine, Fayoum University, Fayoum 63514, Egypt; Laa01@Fayoum.edu.eg; 3 Department of Public Health, Ministry of Health, Kuwait City 12009, Kuwait; malseaidan@moh.gov.kw; 4 Hospital Administration, Farwaniya Hospital, Kuwait City 85000, Kuwait; alrashidi_mb@hotmail.com

**Keywords:** coronavirus disease, healthcare professionals, Middle East, vaccine effectiveness, infectious diseases, public health

## Abstract

Background: Estimating vaccine effectiveness (VE) against severe, acute respiratory syndrome coronavirus 2 (SARS-CoV-2) among healthcare workers (HCWs) is necessary to demonstrate protection from the disease. Between 24 December 2020 and 15 June 2021, we determined the factors associated with vaccine coverage and estimated VE against SARS-CoV-2 infection in HCWs at a secondary hospital in Kuwait. Methods: We extracted sociodemographic, occupational, SARS-CoV-2 infection, and vaccination data for eligible HCWs from the hospital records. Vaccine coverage percentages were cross-tabulated with the HCW factors. Cox regression was used to estimate hazard ratios in vaccinated versus unvaccinated. Results: 3246 HCWs were included in the analysis, of which 82.1% received at least one vaccine dose (50.4% only one dose of ChAdOx1, 3.3% only one dose of BNT162b2, and 28.3% two doses of BNT162b2). However, 17.9% of HCWs were unvaccinated. A significantly lower vaccination coverage was reported amongst female HCWs, younger age group (20–30 years), and administrative/executive staff. The adjusted VE of fully vaccinated HCWs was 94.5% (95% CI = 89.4–97.2%), while it was 75.4% (95% CI = 67.2–81.6%) and 91.4% (95% CI = 65.1–97.9%) in partially vaccinated for ChAdOx1 and BNT162b2, respectively. Conclusions: BNT162b2 and ChAdOx1 vaccines prevented most symptomatic infections in HCWs across age groups, nationalities, and occupations.

## 1. Introduction

The two anti-COVID-19 vaccines (BNT162b2 mRNA [Pfizer, New York, NY, USA; BioNTech, Mainz, Germany] and ChAdOx1 nCoV-19 adenoviral [Oxford-AstraZeneca] are effective in preventing asymptomatic/symptomatic COVID-19, hospitalization, and death according to clinical trials and population-level observational studies [1,2,3,4,5,6]. Both vaccines have been authorized for use in Kuwait. The local BNT162b2 vaccination roll-out started on 24 December 2020, and was followed by the ChAdOx1 roll-out on 3 February 2021.

COVID-19 vaccine effectiveness under ‘real-world’ conditions are important to conduct, not only in the general population, but also in risk-specific groups such as healthcare workers (HCWs). It is well-known that HCWs come in contact with patients either directly or indirectly depending on their occupation, rendering them vulnerable to higher exposure rates to SARS-CoV-2 than the general population. Therefore, they are considered a high-risk group for SARS-CoV-2 infection and, consequently, at risk for disease complications.

Anti-COVID-19 vaccine uptake by HCWs is expected to be high due to their occupational risk. However, early findings showed that the uptake percentage varies between countries. For instance, most HCWs were vaccinated with at least one dose within two to three months from the vaccine roll-out in several countries (e.g., it was 90% in UK; 79% in Israel; 75% in USA, and 73.5% in Spain) [4,7,8,9]. However, in this region, a questionnaire-based study from Saudi Arabia reported that only 33.3% of HCWs enrolled to receive or had already received the vaccine with a greater proportion being females, those of younger age (20–40 years), and those who are Saudi nationals [10].

Recent studies have shown that COVID-19 vaccination reduces the rate of infection among HCWs [4,7,8,11]. In the study from Israel, the incidence rate among BNT162b2 vaccinated HCWs was 3 cases per 10,000 person-days compared to 7.4 cases per 10,000 person-day in unvaccinated [7]. Moreover, a study from United Kingdom (UK) revealed that a single dose of BNT162b2 was effective in reducing SARS-CoV-2 infections (21 days after post vaccination) by 70% among HCWs and 85% after 7 days from receiving the second dose [4]. Furthermore, a study from the United States (US) on two mRNA vaccines (BNT162b2 and Moderna mRNA-1273) conducted on HCWs, full immunization (≥14 days after second dose) was 90% effective in preventing SARS-CoV-2 infections regardless of the symptom’s status, whereas 80% effectiveness was reported in partially immunized HCWs (≥14 days after first dose but before receiving the second dose) [8].

While there are few studies that evaluated mRNA vaccine effectiveness in preventing SARS-CoV-2 infections in HCWs population; there is limited data on both mRNA and non-mRNA vaccine effectiveness in this population. Furthermore, there is very limited published data on vaccine effectiveness among HCWs in the Gulf Cooperation Council countries and most Middle Eastern countries. In one case-series study conducted on 24 HCWs at tertiary care hospitals in Saudi Arabia, authors revealed that only two HCWs (10%) were infected with SARS-CoV2 one week after their second BNT162b2 dose [12]. Therefore, the objective of this retrospective cohort study was to assess the two vaccines (BNT162b2 and ChAdOx1) effectiveness against symptomatic SARS-CoV-2 infection and in relation to HCWs characteristics. We anticipate that the findings of this study will inform HCWs across the country, the region, and internationally, of the importance of vaccination against COVID-19.

## 2. Methods

### 2.1. Study Design and Study Population

The study was a retrospective cohort study among HCWs working at a public secondary hospital in Kuwait. The hospital is a 900-bed facility with multiple medical and surgical specialties including outpatient polyclinics. The original study population was 3673 HCWs (aged ≥ 20 years) working at this hospital. The study started on 24 December 2020 (i.e., the day vaccine roll-out began in Kuwait). HCWs with PCR (polymerase chain reaction)-confirmed SARS-CoV-2 infection before the start of the study were excluded. The study ended on 15 June 2021. The two cohorts we followed were vaccinated and non-vaccinated HCWs. The main outcome of interest was symptomatic SARS-CoV-2 PCR-confirmed infections.

### 2.2. Data Collection

Vaccination data were obtained from the hospital records. The hospital administration staff started collecting vaccination data on 10 January 2021 from all HCWs. The HCWs received their vaccine dose(s) either at this hospital or at different vaccination centers in Kuwait. Nonetheless, the hospital administration staff regularly followed up with all hospital departments and requested specific vaccine-related data on their HCWs including vaccination status (vaccinated or unvaccinated); vaccination date (for first and second dose); vaccine type (BNT162b2 or ChAdOx1); SARS-CoV-2 PCR-confirmed infections (symptomatic or asymptomatic) and the infection date. Additionally, sociodemographic (sex, age, and nationality), occupation setting (e.g., outpatient, inpatient, intensive care), and staff occupation (e.g., doctor, nurse, pharmacist) were available in the hospital records. The sociodemographic and occupation data were matched with collected vaccination data using the civil identification number of the HCWs. The SARS-CoV-2 testing was voluntary on the basis of appearance of COVID-19-like symptoms or being in close-contact with COVID-19 positive case. A HCW was considered COVID-19 symptomatic if he/she had at least one typical disease symptom such as fever, cough, or change in taste or smell. However, the hospital records did not include the range of symptoms but rather classified the infection as either symptomatic or asymptomatic.

The full data were extracted from the hospital records on 15 June 2021. To avoid misclassification of exposure, HCWs with missing vaccination information (e.g., no vaccination date or vaccination type) or missing PCR testing information were excluded from the analysis. Specifically, the analysis excluded 24 HCWs who had a documented SARS-CoV-2 PCR-confirmed infection prior to the study’s starting date and an additional 403 HCWs who had missing, or incomplete, vaccination data or symptom data were also excluded. Hence, a total of 3246 HCWs were eligible for this study.

### 2.3. Outcomes

The primary outcome for the vaccine effectiveness analysis was the SARS-CoV-2 PCR-confirmed infection among unvaccinated or vaccinated at any time during the study (i.e., during the follow-up time) irrespective of symptom status. Infections were described as symptomatic if their symptom status was seven days before or seven days after their PCR positive test date.

The primary outcome for the vaccine coverage analysis was the vaccination status (first or second dose) by vaccine type. Healthcare workers vaccinated with ChAdOx1 had received only one dose by the end of study period. This was due to the delay in ChAdOx1 vaccine shipment to Kuwait that resulted in unavailability of the second dose for the HCWs who were vaccinated in February and March (12-week waiting period between two doses). The ChAdOx1 shipment arrived in Kuwait on 13 June 2021; however, none of HCWs in this study received the second dose by 15 June 2021.

### 2.4. Person-Time AT-Risk

The follow-up of all HCWs started on 24 December 2020, the day vaccine roll-out started in Kuwait. All HCWs had at least one day of follow-up as unvaccinated. For each HCW, the follow-up time (person-time at risk) ended at the earliest of the following three events: the occurrence of the outcome event (SARS-CoV-2 PCR-confirmed infection), vaccination (for unvaccinated), or end of the study period.

### 2.5. Data Analysis

Data were stratified by sociodemographic and occupation factors (i.e., covariates). The covariates were: age group (20–30, 31–40, 41–50, and >50), sex (male or female), nationality (Kuwaiti national and non-Kuwaiti resident), staff group (Administrative or Executive; Nursing or Health-care assistant; Doctor; Specialist Staff; Estates, Porters, or Security; and Pharmacist) and occupation settings (categorized into six groups: (1) office or laboratory, (2) hospital pharmacy, (3) outpatient including radiology, day ward, general practice, or renal dialysis unit, (4) inpatient ward, theatres, emergency department, maternity unit or labor ward, or ambulance, (5) intensive care, and (6) other (e.g., plaster and observational rooms)).

For vaccine coverage analysis, we cross-tabulated three vaccination statuses (unvaccinated; vaccinated with one ChAdOx1 dose; and vaccinated with one or two doses of BNT162b2) with the study’s covariates. The relationship between vaccine coverage status and covariates were assessed via chi-square statistic using STATA software version 16.1 (Stata Corp., College Station, TX, USA). Furthermore, we cross-tabulated SARS-CoV-2 PCR-confirmed infection by vaccination status with the study’s covariates. Similarly, chi-square statistic was used to assess the relationships.

We used retrospective cohort study design to estimate the vaccine effectiveness in the HCWs population after the first and second dose. For the purpose of vaccine effectiveness analysis, the HCWs were defined as unvaccinated (if they had not received any doses of either vaccine), fully vaccinated (if at least 14 days had passed since receiving the second dose of BNT162b2), and partial vaccination (if at least 28 days passed since receiving ChAdOx1 first dose or at least 14 days since receiving BNT162b2 first dose but before receiving the second dose). The BNT162b2 vaccine 13 person-days from receiving the first dose to the partial or full vaccination were excluded from the analysis as at-risk person-time because the immunity was considered indeterminate. Similarly, the ChAdOx1 vaccine 27 person-days after receiving the first dose were excluded. Therefore, the incidence rates were calculated for the: (1) unvaccinated, (2) ≥28 days after receiving ChAdOx1 first dose, (3) ≥14 days after receiving BNT162b2 first dose through receipt of the second dose, and (4) ≥14 days after BNT162b2 second dose. Hazard ratios were estimated using Cox proportional hazards model while accounting for time-varying vaccination status (i.e., receiving first and second dose) as described elsewhere [13]. Hazard ratios of partial vaccination person-days (≥28 days after receiving ChAdOx1 first dose; ≥14 days after receiving BNT162b2 first dose and before second dose) and of full vaccination person-days (≥14 days after BNT162b2 second dose) were calculated and compared to that of unvaccinated person-days. Vaccine effectiveness was calculated as 100% × (1 − hazard ratio). An adjusted vaccine effectiveness model included the covariates individually (i.e., univariate models), and those that were significant at *p* < 0.1 were included in the multivariate model. All analyses were conducted in STATA statistical software.

## 3. Results

There were 3246 HCWs that met the inclusion criteria and included in the analysis. The median age of HCWs was 38 years (IQR = 33–44). Most of HCWs were females (63.4%), aged 31–40 (46.8%), non-Kuwaiti (82.3%), and worked in inpatient wards or ambulance settings (47.3%) as shown in Table 1. Furthermore, 61.2% of HCWs were nursing or health-care assistant staff.

Overall, 82.1% of HCWs received at least one vaccine dose, while 17.9% of HCWs remained unvaccinated by the end of the study. Interestingly, about half of the HCWs (50.4%) received only one dose of ChAdOx1 vaccine; whereas 3.3% received one dose of BNT162b2 vaccine and 28.3% received two doses of BNT162b2 vaccine. Those who received only one dose of BNT162b2 (3.3%) did not receive their second dose because they were SARS-CoV-2 infected after the first dose (only two HCWs) or the study ended before they received it.

The percentage of HCWs classified as partially vaccinated (i.e., ≥28 days after receiving one dose of ChAdOx1 or ≥14 days after receiving BNT162b2 first dose through receipt of second dose) was 50.2% and 2.8%, respectively. However, the percentage of HCWs classified as fully vaccinated (≥14 days after BNT162b2 second dose) was 28.2%.

The characteristics of unvaccinated and vaccinated HCWs by the two types of vaccine are shown in Table 1. Twenty percent of females were unvaccinated compared to 10.6% of males (*p* < 0.001). For the age groups, 28.2% of HCWs aged 20–30 was unvaccinated, significantly higher than other age groups (*p* < 0.001); whereas, within those who received one or two doses of BNT162b2 vaccine, the percentage of vaccinated HCWs in age groups (20–30 and > 50) was higher than that in other age groups (*p* < 0.001). Interestingly, the percentages of unvaccinated Kuwaitis (20.7%) and non-Kuwaitis (17.3%) HCWs were not significantly different (*p* = 0.054); however, within those who received one or two doses of BNT162b2, 60.7% of Kuwaiti HCWs were vaccinated compared to 25.8% for non-Kuwaitis (*p* < 0.001). Among the different occupation settings, 23.3% of HCWs who worked in office or laboratories were unvaccinated, significantly higher compared to the remaining settings (*p* < 0.001) except for ‘other’. As for the HCW staff groups, 31.5% of administrative or executive staff were unvaccinated, significantly higher than other groups (*p* < 0.001). In addition, 58.4% of doctors received one or two doses of BNT162b2 vaccine, which is significantly higher than other staff groups (*p* < 0.001).

SARS-CoV-2 PCR-confirmed infection prevalence with reported symptoms was 7.3% (237/3246) during the study period. There were two additional SARS-CoV-2 PCR-confirmed HCW infections with missing symptomatic status; hence, they were excluded from the analysis. Therefore, all the 237 SARS-CoV-2 PCR-confirmed infections were classified as symptomatic. As shown in Table 2, the infection prevalence was significantly higher among unvaccinated female HCWs (20.8%) compared to 7.1% in those vaccinated with ChAdOx1 and 1.96% in those vaccinated with one or two doses of BNT162b2. Similar findings were observed among male HCWs. The SARS-CoV-2 PCR-confirmed infection prevalence in the different age-groups and by nationality were significantly higher in unvaccinated compared to the vaccinated groups. Furthermore, the infection prevalence was significantly higher across the unvaccinated occupation settings compared to those in vaccinated occupation settings except for “other” where the differences were not significant (*p* = 0.508). The infection prevalence in staff groups were also significantly higher in unvaccinated compared to vaccinated except for pharmacists (*p* = 0.866) (Table 2).

There were 114 SARS-CoV-2 infections during the 90,484 person-days of follow-up in the unvaccinated group, an incidence rate of 126 per 100,000 person-days (Table 3). In the partially vaccinated group, ≥28 days after ChAdOx1 first dose, there were 87 infections (incidence rate of 31.4 per 100,000 person-days). Moreover, in the partially vaccinated group (≥14 days after receiving BNT162b2 vaccine through receipt of second dose), there were two infections (incidence rate of 10.9 per 100,000 person-days). In the fully vaccinated group (≥4 days after BNT162b2 second dose), there were 10 infections (incidence rate of 6.3 per 100,000 person-days).

The estimated, adjusted vaccine effectiveness of fully vaccinated HCWs was 94.5% (95% confidence interval [CI] = 89.4–97.2%). The vaccine effectiveness of partially vaccinated HCWs for ChAdOx1 (≥28 days after one dose) was 75.4% (95% CI = 67.2–81.6%) and for one dose BNT162b2 (≥14 days through receipt of second dose) was 91.4% (95% CI = 65.1–97.9%) (Table 3). The individual covariates (sex, age group, nationality, occupation setting, staff group) were significant predictors; hence, included in the multivariate model. However, these covariates were not significant (*p* > 0.05) in the adjusted vaccine effectiveness multivariate model. We kept the sociodemographic variables (sex, age group, and nationality) in the adjusted model and compared the change between unadjusted and adjusted models. The change in vaccine effectiveness point estimates were <1% between unadjusted and adjusted models.

## 4. Discussion

This retrospective cohort study, conducted between 24 December 2020 and 15 June 2021 (i.e., 173 days) at a secondary hospital in Kuwait, shows that full vaccination (i.e., immunization) via BNT162b2 is highly effective in preventing symptomatic COVID-19 among this HCW population. Furthermore, ChAdOx1 one dose was relatively effective (Table 3).

Vaccine coverage with at least one dose among HCWs after 173 days (about 5.8 months) was 82.1% of HCWs including 28.3% who received two doses. However, there were 17.9% of HCWs unvaccinated by the end of the study, which is a concern. Healthcare workers have been given the priority for vaccination in Kuwait as in most countries; therefore, efforts are needed to better understand reasons for vaccine hesitancy in this high-risk exposure group. Other studies have reported that most HCWs were vaccinated with at least one dose within two to three months of vaccine roll-out (i.e., 90% in UK; 79% in Israel; 75% in USA, 73.5% in Spain) [4,7,8,9].

There were significant differences in vaccine coverage by demographics, occupation setting, and staff group. Similar differences by factors such as age, sex, ethnicity, and occupation have been reported in other studies [14,15,16]. The differences in vaccine coverage that we reported among this population highlights the importance of an equitable vaccination program to all HCWs in Kuwait. The main challenge to vaccine distribution during the first few months of the vaccine program was the shortage in vaccine supply to Kuwait. It is well-known that vaccine nationalism, where countries prioritize their own citizens for vaccination, is a challenge for vaccine equity and for vaccine access to communities across the world [17]. However, as vaccine production increased in mid-2021, vaccine became more accessible; hence, vaccine distribution improved across different communities in Kuwait.

Vaccine effectiveness of full vaccination with two doses of BNT162b2 was 94.5% (95% CI = 89.4–97.2%) against symptomatic PCR-confirmed SARS-CoV-2 infection, whereas it was 75.4% (95% CI = 67.2–81.6%) for ChAdOx1 single dose. These findings are consistent with those from other population-level studies that estimated vaccine effectiveness against SARS-CoV-2 infection (symptomatic and/or asymptomatic) among HCWs [4,7,8,11] and those from vaccine phase III trials [2,18]. For instance, in a study from the US CDC, the authors reported that vaccine effectiveness against SARS-CoV-2 infection among HCWs with full immunization (≥14 days after BNT162b2 second dose) was 90% (95% CI = 68–97%) [8]. In other studies on HCWs, BNT162b2 vaccine effectiveness against SARS-CoV-2 infection (≥7 day post second dose) was 85% (95% CI = 74–96%) in the UK [4],90.6% (Cis were not provided) ≥7 day post second dose in Spain [9], 94.2% (CI: 88.5%-98.1%) after second dose in Greece [19], and 85% (95% CI = 71–92%) 15–24 days after second dose in Israel [7]. In another study from Italy, authors reported that BNT162b2 vaccine among HCWs reduced COVID-19 incidence rate and symptom durations significantly after 13–21 days from a single dose administration [20]. The main difference between these studies and ours is that HCWs were regularly tested for SARS-CoV-2 infection (active surveillance), while in our study it was based on reports of PCR-confirmed infections by the HCWs to the hospital management (passive surveillance). Nonetheless, it was mandatory for the HCWs to report if they were in close contact with a positive case or have tested positive via a nasopharyngeal PCR test.

Our findings highlight the effectiveness of vaccine in reducing the risk of symptomatic infection among HCWs across sociodemographic factors (sex, age, and nationality) and across occupation setting and staff group. Importantly, reducing infection rate among HCWs via vaccination is critical to protect their health and lower the transmission risk to their contacts (coworkers and patients), as well as to the public [21].

The partial vaccination (≥28 days after ChAdOx1 one dose) provided about 75% protection. This result is similar to the Phase III clinical trial results for this vaccine [18,22]. However, to the best of our knowledge, there is no estimate on vaccine effectiveness of ChAdOx1 in a HCW population. BNT162b2 partial vaccination (≥14 days after first dose but before the second dose) also provided a high level of protection from infection in HCWs in this study; however, this was limited by the relatively short at-risk person-time. Recent studies showed that partial vaccination among HCWs in the U.S. (≥21 days after BNT162b2 first dose) had vaccine effectiveness of 80% (95% CI = 59–90%) and 72% (95% CI = 58–86%) in a study from UK [4,8]. Both studies were based on a regular SARS-CoV-2 testing program. Moreover, a study from Israel reported that one dose BNT162b2 provided 60% (95% CI = 38–74%) vaccine effectiveness against confirmed SARS-CoV-2 infection based on hospital records (passive reporting) [7]. It is worth mentioning that SARS-CoV-2 B.1.1.7 (Alpha) variant was detected in Kuwait in January 2021 and could have been the dominant variant during the study period; however, there are no available data in Kuwait to determine the percentage and distribution of infections with this variant. Moreover, the B.1.617.2 (Delta) variant was reported in Kuwait in late June 2021 (after the study ended); hence, it did not confound the vaccine effectiveness results.

This study has several limitations. First, the study was based on one public secondary hospital and might not be generalizable to HCWs in other public hospitals in Kuwait. However, this hospital is one of the major healthcare facilities in Kuwait and serves over a quarter of the country’s population. Second, the identification of HCWs SARS-CoV-2 PCR-confirmed infections was based on passive reporting to the hospital management due to lack of active laboratory surveillance. However, it was/is required by all HCWs to report PCR-confirmed infections to their upper management within each hospital’s department. Furthermore, underreporting PCR-confirmed infections might underestimate the ‘actual’ number of infections regardless of vaccination status; if this disproportionately impacted those who were unvaccinated compared to those who were vaccinated, this could overestimate vaccine effectiveness. Third, vaccine effectiveness estimates for partial immunity (≥14 days after BNT162b2 first dose through receipt of second dose) had wide confidence intervals that likely due to the low number of PCR-confirmed infections reported. Fourth, the differences in vaccine coverage by some of the HCW demographics (e.g., nationality), occupation setting, and staff group might have affected the vaccine effectiveness estimates since some receipts have received vaccination earlier and/or of a different type compared to others. However, this was part of the nature of the vaccine coverage program where variability could exist in receiving the vaccine (type and time). Fifth, SARS-CoV-2 infection incidence rates in Kuwait’s general population was variable during the study time, which might have impacted the overall vaccine effectiveness estimates. However, if there was an impact, it would occur for both vaccinated and unvaccinated groups.

## 5. Conclusions

The vaccine effectiveness of both BNT162b2 and ChAdOx1 COVID-19 vaccines in HCWs under ‘real-world’ conditions demonstrated that vaccine is effective in preventing most symptomatic infection across age groups, nationalities, occupation setting, and staff groups. A significant proportion (17.9%) of HCWs were unvaccinated despite the vaccine accessibility. Efforts are needed to better understand reasons for HCW vaccine hesitancy. Although vaccination is highly effective against infection, hospitalization, and mortality as shown in other studies, it is important for HCWs to continue to exercise physical distancing, wear personal protective equipment while in contact with patients, and follow other infection control and prevention measures.

## Figures and Tables

**Table 1:** Characteristics of vaccinated and unvaccinated HCWs (*n* = 3246) and factors associated with ChAdOx1 or BNT162b2 coverage at a major secondary hospital in Kuwait, 24 December 2020–15 June 2021.

Characteristics	Categories	Total No.	Unvaccinated (Row %)	Vaccinated with One Dose Only (ChAdOx1) (Row %)	Vaccinated with One or Two Doses (BNT162b2) ^a^ (Row %)
Sex	Female	2075	20.0	50.9	27.1
	Male	1171	10.6	48.8	40.7
	*p*-value ^b^		<0.001	0.267	<0.001
Age groups	20–30	443	28.2	30.9	40.9
	31–40	1518	19.7	55.0	25.3
	41–50	869	12.5	55.6	31.9
	>50	416	11.3	41.6	47.1
	*p*-value		<0.001	<0.001	<0.001
Nationality	Kuwaiti	575	20.7	18.6	60.7
	Non-Kuwaiti	2671	17.3	56.9	25.8
	*p*-value		0.054	<0.001	<0.001
Occupation setting ^c^	Offices and laboratory (lower risk)	446	23.3	36.8	39.9
	Patient facing non-clinical	170	15.3	47.1	37.7
	Outpatient	618	16.7	45.5	37.9
	Inpatient wards and ambulance	1536	18.4	53.4	28.2
	Intensive Care (Higher risk)	407	11.6	58.2	30.2
	Other	69	24.6	66.7	8.7
	*p*-value		<0.001	<0.001	<0.001
Staff group	Administrative or executive	54	31.5	24.1	44.4
	Nursing or health-care assistant	1985	17.4	59.5	23.1
	Doctor	541	11.5	30.1	58.4
	Specialist Staff	569	24.1	40.8	35.2
	Estates, porters, or security	15	0	100	0
	Pharmacist	82	22	29.3	48.8
	*p*-value		<0.001	<0.001	<0.001
Total (%)		3246	581 (17.9)	1636 (50.4)	1029 (31.7)

^a^ Total vaccinated includes 108 HCWs who received one BNT162b2 vaccine dose and 921 who received two BNT162b2 vaccine doses. ^b^ *p*-values (comparing the column percentages of vaccinate status by sociodemographic, occupation setting, and staff group categories were calculated using Pearson’s chi-square test (cells with ≥5 observations) or Fisher’s exact test (cells with <5 observations) in STATA statistical software ver. 16.1 (Stata Corp., College Station, TX, USA). ^c^ Occupation setting categories were: 1: office or laboratory; 2: Hospital pharmacy, 3: outpatient including radiology, day ward, general practice, or renal dialysis unit; 4: inpatient ward, theatres, emergency department, maternity unit or labor ward, or ambulance; 5: intensive care; and 6: other (e.g., plaster and observational rooms).

**Table 2:** Prevalence of SARS-CoV-2 PCR–confirmed infections in HCWs (*n* = 3246) received BNT162b2 or ChAdOx1 COVID-19 vaccines at a major secondary hospital in Kuwait, 24 December 2020–15 June 2021.

Characteristics	Categories	No. Unvaccinated *n* (Infection %)	Vaccinated with One Dose Only (ChAdOx1) *n* (Infection %)	Vaccinated with One or Two Doses (BNT162b2) *n* (Infection %) ^a^	*p*-Value ^b^
Sex	Female	456	1057	562	<0.001
		(20.8)	(7.1)	(2.0)	
	Male	124	571	476	<0.001
		(15.3)	(4.9)	(1.9)	
Age groups	20–30	125	137	181	<0.001
		(21.6)	(9.5)	(2.8)	
	31–40	299	835	384	<0.001
		(16.7)	(6.6)	(0.8)	
	41–50	109	483	277	<0.001
		(25.7)	(6.0)	(2.2)	
	>50	47	173	196	<0.001
		(19.2)	(3.5)	(3.1)	
Nationality	Kuwaiti	119	107	349	<0.001
		(16.8)	(7.5)	(2.0)	
	Non-Kuwaiti	461	1521	689	<0.001
		(20.4)	(6.3)	(1.9)	
Occupation setting ^c^	Offices and laboratory (lower risk)	104	164	178	<0.001
		(26.9)	(9.2)	(2.8)	
	Patient facing non-clinical	26	80	64	<0.001
		(19.2)	(5.0)	(3.1)	
	Outpatient	103	281	234	<0.001
		(12.6)	(3.2)	(2.1)	
	Inpatient wards and ambulance	283	820	433	<0.001
		(19.8)	(6.5)	(0.9)	
	Intensive Care (Higher risk)	47	237	123	<0.001
		(21.3)	(7.2)	(3.3)	
	Other	17	46	6	0.508
		(11.8)	(10.9)	0.0	
Staff group	Administrative or executive	17	13	24	<0.001
		(23.5)	(15.4)	(4.2)	
	Nursing or health-care assistant	346	1181	458	<0.001
		(20.5)	(6.4)	(1.3)	
	Doctor	62	163	316	<0.001
		(19.4)	(5.5)	(1.9)	
	Specialist Staff	137	232	200	<0.001
		(19.0)	(6.5)	(2.5)	
	Estates, porters, or security	0	15	0	-
		0.0	0.0	0.0	
	Pharmacist	18	24	40	0.866
		(5.6)	(8.3)	(6.1)	

^a^ Total vaccinated includes 108 HCWs who received one BNT162b2 vaccine dose and 921 who received two BNT162b2 vaccine doses. ^b^ *p*-values (comparing the row percentages of vaccinate status by sociodemographic, occupation setting, and staff group categories were calculated using Pearson’s chi-square test (cells with ≥5 observations) or Fisher’s exact test (cells with <5 observations) in STATA statistical software ver. 16.1 (Stata Corp., College Station, TX, USA).“-“ because of lack of enough data for chi-square statistic to test relationship. ^c^ Occupation setting categories were: 1: office or laboratory; 2: Hospital pharmacy, 3: outpatient including radiology, day ward, general practice, or renal dialysis unit; 4: inpatient ward, theatres, emergency department, maternity unit or labor ward, or ambulance; 5: intensive care; and 6: other.

**Table 3:** Effectiveness of ChAdOx1 and BNT162b2 COVID-19 vaccines against SARS-CoV-2 symptomatic infection among HCWs (*n* = 3246) at a major secondary hospital in Kuwait, 24 December 2020–15 June 2021.

COVID-19 Vaccination Status	Total Person Time (Days)	Number of PCR Positives	Incidence Rateper 100,000 Person-Days	Unadjusted Vaccine Effectiveness % (95% CI) ^a^	Adjusted Vaccine Effectiveness % (95% CI) ^a,b^
Unvaccinated	90,367	114	126.2	Reference	Reference
Partially vaccinated					
≥28 days after receiving ChAdOx1 first dose only ^c^	159,423	87	54.6	75.5 (67.6–81.5)	75.4 (67.2–81.6)
≥14 days after receiving BNT162b2 first dose through receipt of second dose	7196	2	27.8	91.6 (65.9–97.9)	91.4 (65.1–97.9)
Fully vaccinated					
≥14 days after BNT162b2 second dose	90,015	12	13.3	95.1 (90.6–97.4)	94.5 (89.4–97.2)

^a^ Vaccine effectiveness was estimated using a Cox proportional hazards model accounting for time-varying immunization status in STATA statistical software ver. 16.1 (Stata Corp., College Station, TX, USA). ^b^ Hazard ratio is adjusted for age, sex, and nationality. ^c^ Participants received first dose of ChAdOx1 but had not received second dose by the end of the study period. PCR: polymerase chain reaction; CI: confidence interval.

## Data Availability

The data supporting this study will be made available upon reasonable request.
